# C-KIT Expression in Orbital Cavernous Venous Hemangiomas

**DOI:** 10.3390/biom11081199

**Published:** 2021-08-12

**Authors:** Mizhir Atallah, Natalia Edison, Esther Levi, Irit Elmalah, Daniel Briscoe

**Affiliations:** 1 Ophthalmology Department, Emek Medical Center, Afula 18101, Israel; ATALLAHMI@clalit.org.il; 2 Laboratory of Ophthalmic Research, Emek Medical Center, Afula 18101, Israel; ester_le1@clalitt.org.il; 3 The Tissue Diagnostics and Cancer Research Institute, Emek Medical Center, Afula 18101, Israel; natalia_ed@clalit.org.il (N.E.); elmalaj_ir@clalit.org.il (I.E.)

**Keywords:** orbital cavernous hemangioma, c-KIT, immunohistochemistry

## Abstract

Orbital (slow flow) cavernous venous hemangiomas (OCVH) are the most common benign orbital tumors in adults. The c-KIT is a tyrosine kinase receptor, which is expressed on several types of cells, is thought to play a key role in tumor pathogenesis. The purpose of this study was to evaluate the presence of the receptor c-KIT in OCVH. Our retrospective study examined 16 orbital cavernous venous hemangiomas from 16 cases operated on between 2006–2016 at Emek Medical Center. The mean tumor size was 18.4 mm. Symptoms appeared between 6 months and 22 years before operation. All specimens were analyzed for the c-KIT receptor through immunohistochemistry. The c-KIT was expressed by the endothelium in all 16 preparates. Staining was strong in two cases, moderate in six, and weak in eight cases, with no statistically significant correlation between staining and tumor size (*p* = 0.69) or the symptom duration (*p* = 0.15). We conclude that c-KIT may play an important role in the pathogenesis of OCVH. This pilot study is significant in that tumor-targeted therapy such as Imatinib Mesylate and Sunitinib may have a role in treating surgically complicated cases located in the orbital apex. A large multicenter collaborative study is necessary to examine the role of c-KIT in OCVH.

## 1. Introduction

Orbital (slow flow) cavernous venous hemangiomas (OCVHs) are the most common benign orbital tumors in adults and constitute between 3–9% of orbital masses [1,2]. They are benign tumors of the orbit and affect both men and women with a mean age of 40–48 years. Usually, they have a typical clinical presentation characterized by slowly developing proptosis, diplopia, a reduction of visual acuity, and the restriction of the visual field. Surgery to remove OCVHs presents a more significant risk of vision loss, especially when the mass is located in the orbital apex. Although they are well characterized clinically [3], their mechanisms of pathogenesis are still elusive. In recent years, however, several studies have investigated the expression of different receptors and markers in OCVHs [4,5,6,7] and have thrown some light to their characteristics.

The c-KIT is a tyrosine kinase receptor which is expressed on hematopoietic stem cells and on lung mast cells [8]. It has also been described on different cells including eosinophils [9], basophils [10], human vascular smooth muscle cells [11], epithelial cells [12], and human umbilical vein endothelial cells [13,14]. Activation of the c-KIT may result in several cellular responses including proliferation, differentiation, adhesion, survival, programmed cell death, among others. c-KIT is thought to be involved in tumor pathogenesis, including hematologic neoplasia [15], gastrointestinal stromal tumors [16], mastocytosis, seminoma, sarcoma, among others [17,18,19].

Although several publications have examined the c-KIT in hemangiomas from the body in general, to the best of our knowledge, there are no publications regarding the presence of the c-KIT in Cavernous Venous Hemangiomas or in other types of vascular tumors specifically involving the orbit. The purpose of the present study was to examine the presence of c-KIT in OCVH. Several tyrosine kinase inhibitors, including Imatintib, Sunitinib, Gefitinib, and Erlotinib, are currently being used in the treatment of gastrointestinal stromal tumors, lung cancer, esophageal cancer, pancreatic cancer, and adenocarcinoma. The presence of c-KIT receptors in OCVH could open up a whole new non- surgical approach to the treatment of these vision threatening orbital tumors.

## 2. Materials and Methods

All of the experimental protocols of this study were approved by the EMC Ethics Committee review board. We confirm that study number for this study was EMC0086-11. Methods were conducted in accordance with ICH-GCP and Ministry of Health guidelines and was approved by the EMC Ethics Committee review board. Informed consent was waived based on the permission of the EMC Ethics Committee review board, as the research was retrospective and presented no threat to the rights and welfare of the research subjects. The information is not sensitive in nature, and the data are derived from specimens that had previously been excised from the orbit where clinically indicated.

A total of sixteen orbital cavernous venous hemangioma specimens were collected from patients who had been operated on at our medical center between January 2006 and January 2016. Demographics, clinical, and ocular histories were recorded for each of the 16 patients. Tissue was routinely fixed in formalin and was embedded in paraffin. The paraffin sections were cut at 3-μm and were routinely stained with Hematoxylin and Eosin. The diagnosis of cavernous hemangioma for each specimen was reconfirmed by a senior pathologist (I.E.).

For staining of c-KIT, formalin-fixed 3-μm paraffin-embedded sections were mounted on Surgipath™ X-tra™ Adhesive precleaned micro slides (Leica microsystems, Buffalo Grove, IL, USA) and were processed using an automated immunostainer (Benchmark-ulra, Ventana Medical System, USA.). An anti-c-KIT (CD117) (DAKO, A4502, 1:200 concentration) was used as a primary antibody. Visualization of the bound primary antibodies was performed using the I-View DAB detection KIT (Ventana Medical System). Sections were then counterstained with Gill’s hematoxylin, dehydrated, and mounted for microscopic examination. Gastrointestinal stromal tumor served as a positive control. The intensity of the staining was scored as follows: +3, strong; +2, moderate; +1, weak;-negative.

## 3. Results

**Figure 1:**
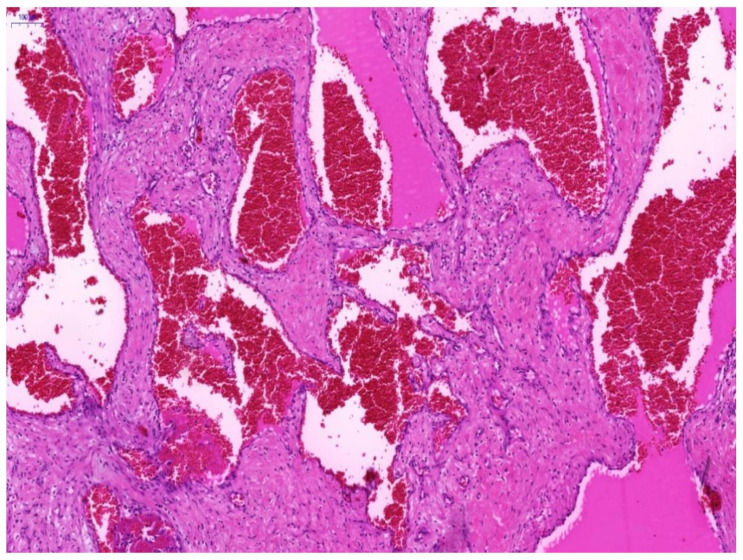
Photomicrograph of a specimen of orbital cavernous hemangioma. Hematoxylin-Eosin staining showed a dilated vascular cavity lined with flattened endothelium and a thick vascular wall. Representative image is presented (magnification ×300. Scale bar 100 μm).

Histologically, OCVH was identified by large vessels filled with blood cells and lined with flattened endothelium (Figure 1).

**Figure 2:**
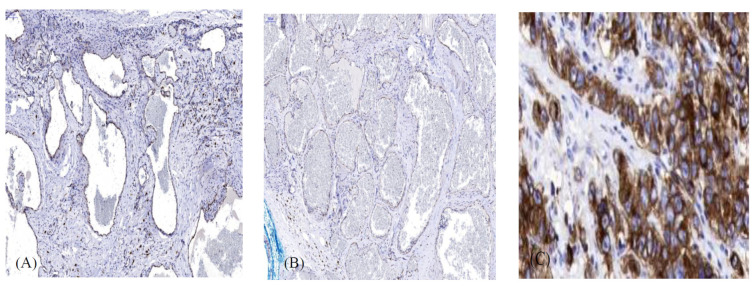
(**A**). Representative cavernous hemangioma with strong immunohistochemical staining positivity for the c-KIT receptor in endothelial cells. (**B**). Representative cavernous hemangioma with weak immunohistochemical staining positivity for the c-KIT receptor in endothelial cells. (**C**). Gastrointestinal stromal tumor served as a positive control. (magnification × 300. Scale bar 100 μm).

All the cases were c-KIT positive with some disparity in the strength of staining. There were two cases with strongly positive staining, six cases with moderate staining, and eight with weak staining. Table 1 and Figure 2A,B show the results of the c-KIT staining. c-KIT was clearly shown to be expressed by the endothelial cells.

Figure 2C shows a comparison to the positive control of a gastrointestinal stromal tumor. Clinical features of the patients are presented in Table 2. Patients were 7–78 years of age (mean 39.9 y), with a female to male ratio of 1:1. The same ratio of 1:1 was also observed regarding the location of the OCVHs in the left or right orbit. The main symptoms at presentation were visual disturbances (8), proptosis (7), and proptosis and ptosis (1). However, 14 patients were found to have proptosis at clinical examination. There were two cases that involved the anterior orbit and the effected eyelid position without proptosis. In the first case, the mass protruded in the upper eyelid, causing mild ptosis. In the second case, the mass was visible in the conjunctival fornix and protruded through the lower eyelid, pushing it up. The symptom duration from onset until the time of the surgery was between 6 months and 22 years. In some cases, there was a small lesion causing no visual impairment. Some of these cases were followed until they began to effect visual function. There were 11 patients who had symptoms between 6 months and 2 years before surgery. There were two cases that had symptoms and that were followed for 4 years, and there were three cases that had symptoms and that were followed between 10 and 22 years until eventually undergoing surgery. In seven cases, the tumor was intraconal, and in nine cases, the tumor was extraconal. The tumor size (maximal) ranged from 8 mm to 28 mm (mean 18.4 mm).

Correlation analysis between c-KIT staining and tumor size or duration of symptoms were not statistically significant (*p* value = 0.6912 and 0.1474 respectively, analyzed using GraphPad software).

## 4. Discussion

In the present, study we have demonstrated the expression of c-KIT in orbital cavernous hemangiomas through immunohistochemical staining. This is the first report of the presence of c-KIT in the endothelial cells of these tumors. The critical roles that c-KIT may play in different functions of the cells and in the pathogenesis of numerous tumors led us to consider studying a potential role in OCVH. Thus, we designed and conducted our study in order to determine whether or not c-KIT is expressed in OCVH and to clarify which cells express it. In our study, which presents the largest series of immunohistochemically studied OCVH in the literature, all the of the 16 cases were positive for c-KIT staining. There was some variation in the strength of the stain between cases according to our protocol. c-KIT was clearly shown to be expressed by endothelial cells, with positive staining in two cases, moderate staining in six, and weak staining in eight cases (Figure 2A,B). Staining disparities between samples might be attributed to methodological causes or to different levels of protein expression. Regarding methodology, we were careful to use standardized techniques for the handling and preparation of the specimens in addition to a uniform staining protocol. However, several explanations might be suggested for real differences in protein expression, including the proliferative capacity of the tumor, the tumor size, the age of patient, and other proteins that might affect, directly or indirectly, the expression of c-KIT. In our study, we could not prove any correlation between the tumor size or the age of patients and the staining strength (data not shown).

Although it is desirable to confirm the immunohistochemical results by proteomic techniques, such as Western blotting, this requires fresh tissue in a tumor that is relatively rare, with only a limited number of cases appearing annually in each center where orbital surgery is performed. Proteomic techniques can also be technically challenging, as other tissue contents, including mast cells, necessarily contribute to the c-KIT protein of the tissue extracts. The use of proteomics and molecular sequencing for the investigation of c-KIT could be the subject of a future prospective multicenter collaborative study using fresh tissue from numerous centers.

Melanoma of the skin and uvea behave differently, and it may be that cavernous hemangiomas in the orbit have different characteristics than cavernous hemangiomas of other parts of the body [20]. Most studies examining c-KIT in angiomas were not specific to cavernous venous hemangiomas and were not from any specific part of the body [21,22,23]. Our study specifically examined cavernous venous hemangiomas of the orbit.

In recent years, several studies characterizing OCVH immnunohistochemically have been published. Di Tommaso et al. [4] have investigated the presence of the smooth muscle markers, including smooth muscle actin (SMA) and desmin, and the sex steroid receptors, progesterone (PR) and estrogen (ER) receptors, in 12 cases of OCVH. They found that the SMA and desmin were localized in the spindle cells of the vascular walls of all 12 cases and that PR was also observed in all 12 cases. ER was negative in all cases. Nagasaka et al. [5] investigated the involvement of several angiogenic factors and their receptors in the growth of nine cases of OCVH. They found that all of the cases were positive for the vascular endothelial growth factor (VEGF). However, another study by Bernatchez and colleagues [24] found that while the basic fibroblast growth factors (bFGF) and the VEGF receptor flk-1, which produce a positive proliferation signal, were expressed in hemangioma, there was no expression of the VEGF receptor flt-1, which negatively regulates endothelial proliferation [25].

Gupta et al. [6] investigated 11 cases of OCVH with respect to proliferative capacity and receptor expression. They found that the proliferating cell nuclear antigen was positive in 10 out of the 11 cases. The proliferating cell nuclear antigen is considered to be an auxiliary protein for DNA polymerase δ during the S phase and shows positive a correlation with proliferating endothelial cells [26]. The BcL2, an anti-apoptotic protein, was positive in eight cases. VEGF and PR were each weakly positive in three cases. All of the cases were negative for Mib-1, a monoclonal antibody developed against the Ki-67 protein, which is a proliferation antigen [27], for D2-40, which is a marker of lymphatic endothelium [28] and for ER. Finally, Osaki et al. [7] conducted a comparative immunohistochemical study of infantile hemangioma and OCVH in adults, using different stains for the GLUT-1, endothelial and lymphatic markers, SMA, desmin, and Ki-67. They found no overlapping staining patterns and demonstrated the architectural differences between the two kinds of lesions.

Through immunohistochemical staining, several publications have addressed the presence of c-KIT in normal and transformed human vascular tissues. Lammie A et al. [21] reported c-KIT negativity in normal human endothelia of arteries, veins, and capillaries. Miettinen M and colleagues [22] have investigated c-KIT expression in malignant and benign vascular tumors in addition to fetal tissues. They found that immunohistochemically, more than half of angiosarcomas and the minority of Kaposi sarcoma express c-KIT, whereas this receptor was not detected in benign vascular tumors, with the exception of occasional infantile capillary hemangiomas. Their series included only five cavernous hemangiomas, and it is not stated whether they were from the orbit or from another body location. Another study by Liu L [23] et al. demonstrated that only three out of ten hemangiomas had weak c-KIT positive staining. Similarly, no cases were reported to be from the orbit.

OCVH is considered as a benign, slow growing tumor. It is traditionally viewed in literature as hamartoma with low proliferative capacities and is attributed to capillary proliferation followed by venous differentiation [3]. An alternative theory suggests that these are vascular malformations that condense into a well-circumscribed tumor [29]. The pathogenesis and origin of this tumor are still elusive with no conceptual consensus in literature. Terminological differences regarding OCVH and other orbital vascular lesions still exist.

Orbital cavernous hemangiomas, although benign, can be sight threatening depending on their size and location. They can cause gradual compression of the optic nerve as they grow and increase in size. A follow-up of patients with no optic nerve compression is possible, but definitive treatment is surgical removal of the tumor with the consequent risks to sight. The risk of visual damage or diplopia from damage to the extraocular muscles or their innervation during surgery is far greater when the hemangiomas are located in the orbital apex. Apical cavernous hemangiomas in the orbit are often firmly attached to the periosteum, providing a greater surgical challenge and demanding a highly skilled and experienced orbital surgeon with a multidisciplinary team. In these cases, medical treatment could potentially be far less risky than surgery and could provide a better treatment option.

The c-KIT tyrosine kinase receptor is considered to be a protooncogene found on human chromosome 4q11–q12 and on murine chromosome 5 [30]. The binding of its ligand, the stem cell factor [31], leads to the activation of multiple pathways, including phosphatidyl-inositol-3 (PI3)-kinase, phospholipase C (PLC)-γ, Src kinase, Janus kinase (JAK)/Signal Transducers, and the Activators of Transcription (STAT) and mitogen activated protein (MAP) kinase pathways. The resulting cellular responses may include proliferation, differentiation, adhesion, survival, programmed cell death, among others. The cellular response, however, is primarily determined to be by cell type and the environment.

Although loss-of-function mutations of c-KIT have not been reported in humans, the gain of function mutations can result in the development of different tumors. In fact, the first descriptions of the role of c-KIT as a cause of human tumors were reported in hematologic [15] and gastrointestinal stromal tumors [16]. Since then, additional reports have been published, and the mutated c-KIT has been found in mastocytosis, seminoma, sarcoma, among others [17,18,19]. Moreover, the expression of c-KIT was immunohistochemically shown in germinoma tumors of the brain [32]. The role of c-KIT in tumors is highlighted by the reports that treatment with its inhibitors, the Imatinib Mesylate (Glivec) and Sunitinib (Sutent) (targeted cancer therapy), result in the inhibition of the tumor cells of some cancers [33,34,35,36,37,38,39]. It has long been theorized by pathologists that hemodynamic disturbance including thrombosis or bleeding in the cavernous hemangioma may cause the mass to suddenly expand.

Orbital (slow flow) cavernous venous hemangiomas have been well classified in previous publications. [40,41,42,43,44] However Rootman et al. [45] found that thrombosis, acute or chronic, was found in each specimen of their series. Acute fibrin clots were noted in 90% of samples. About fifty percent of the time, there were between one and five channels with acute thrombosis per specimen.

Thrombin has been shown to mediate mast cell adhesion through the activation of G(i) proteins, phosphoinositol 3-kinase, protein kinase C, and mitogen-activated protein kinase pathways [46]. Furthermore, in melanoma cells, up-regulation of the thrombin receptor PAR-1 and the matrix metalloproteinase (MMP)-2, and down regulation of the c-KIT were found to be crucial for the metastatic progression of these cells [47]. This may explain why the cavernous hemangiomas of the orbit can suddenly expand through proliferation of vascular channels and progressive ectasia [3]. 

In conclusion, our work demonstrates the presence of the c-KIT in Cavernous venous hemangiomas of the orbit. We believe that this study is significant in that a potential promising medical treatment option may be possible using tyrosine kinase inhibitors which are already available. In recent years, a number of tyrosine kinase inhibitors were developed including Imatintib, Sunitinib, Gefitinib, and Erlotinib. These inhibitors were proved effective in treatment of gastrointestinal stromal tumors, lung cancer, esophageal cancer, pancreatic cancer and adenocarcinoma. However further multicenter studies are needed to examine a much larger number of specimens using the same protocol and to explore the mechanism and role of c-KIT in the pathogenesis of OCVH, including c-KIT signal transductions, molecular testing for c-KIT mutations and others. Understanding the pathogenesis mechanisms of OCVH might pave the way for non-invasive treatment modalities such as tumor-targeted therapy. Furthermore, investigation of the expression of C KIT in Cavernous Hemangioma of other specific anatomical locations such as brain or liver will need to be carried out. Should mutated-C KIT be expressed in Cavernous Hemangiomas of the brain and liver, the potential treatment effect could have a far great impact on modern medicine.

## Figures and Tables

**Table 1:** Summary of c-KIT staining.

Case	Staining Score
1	2
2	2
3	3
4	1
5	2
6	1
7	2
8	1
9	1
10	1
11	3
12	1
13	1
14	1
15	2
16	2

**Table 2:** Summary of clinical features of the OCVH cases. Proptosis was measured using exophthalmometer and was defined as a globe displacement of ≥2 mm compared to the other side.

Case	Age Range	Sex	Tumor Side	Duration of Symptoms (yrs)	Symptom of Onset	Proptosis	Location	Tumor Size (mm)
1	70′s	M	RT	1	proptosis + ptosis	V	Extraconal	12 × 10 × 7
2	50′s	F	LT	2	proptosis	V	Intraconal	15 × 25×10
3	30′s	M	LT	10	proptosis	V	Intraconal	15 × 20 × 25
4	30′s	M	LT	1.5	Visual disturbance	NO	Extraconal	8 × 5 × 7
5	50′s	F	RT	1	Visual disturbance	V	Intraconal	20 × 20
6	Under10	F	RT	4	Visual disturbance	V	Extraconal	18 × 10
7	40′s	F	LE	1	Visual disturbance	V	Extraconal	15 × 10 × 8
8	20′s	F	LE	22	proptosis	V	Intraconal	15 × 15 × 10
9	30′s	M	RT	1	proptosis	V	Intraconal	25 × 15 × 10
10	40′s	F	LE	4	proptosis	V	Extraconal	25 × 15 × 15
11	60′s	M	LE	0.5	proptosis	V	Intraconal	20 × 15 × 10
12	40′s	M	RE	11	Visual disturbance	V	Extraconal	28 × 8
13	20′s	F	RE	1	Visual disturbance	NO	Extraconal	8 × 5 × 3
14	60′s	M	RE	2	proptosis	V	Extraconal	20 × 15 × 10
15	50′s	F	LE	1	Visual disturbance	NO	Extraconal	10 × 10 × 8
16	30′s	M	RE	2	Visual disturbance	V	Intraconal	18 × 15 × 15
